# Modulation of K_V_4.3-KChIP2 Channels by IQM-266: Role of DPP6 and KCNE2

**DOI:** 10.3390/ijms23169170

**Published:** 2022-08-15

**Authors:** Angela de Benito-Bueno, Paula G. Socuellamos, Yaiza G. Merinero, Pilar Cercos, Carolina Izquierdo, Miguel Daniel-Mozo, Irene Marín-Olivero, Angel Perez-Lara, Juan A. Gonzalez-Vera, Angel Orte, Armando Albert, Mercedes Martin-Martinez, Marta Gutierrez-Rodriguez, Carmen Valenzuela

**Affiliations:** 1 Instituto de Investigaciones Biomédicas “Alberto Sols” (CSIC-UAM), 28029 Madrid, Spain; 2 Instituto de Química Médica (IQM-CSIC), 28029 Madrid, Spain; 3 Instituto de Química Física Rocasolano, Consejo Superior de Investigaciones Científicas (IQFR-CSIC), 28006 Madrid, Spain; 4 Nanoscopy-UGR Laboratory, Departamento de Fisicoquímica, Unidad de Excelencia de Química Aplicada a Biomedicina y Medioambiente, Facultad de Farmacia, Campus Cartuja, Universidad de Granada, 18071 Granada, Spain; 5 Department of Neurobiology, Max Planck Institute for Multidisciplinary Sciences, 37077 Göttingen, Germany; 6 Spanish Network for Biomedical Research in Cardiovascular Research (CIBERCV), Instituto de Salud Carlos III, 28029 Madrid, Spain

**Keywords:** K_V_4 channels, KChIP2, DPP6, KCNE, KChIP2 ligand

## Abstract

The transient outward potassium current (*I*_tof_) is generated by the activation of K_V_4 channels assembled with KChIP2 and other accessory subunits (DPP6 and KCNE2). To test the hypothesis that these subunits modify the channel pharmacology, we analyzed the electrophysiological effects of (3-(2-(3-phenoxyphenyl)acetamido)-2-naphthoic acid) (IQM-266), a new KChIP2 ligand, on the currents generated by K_V_4.3/KChIP2, K_V_4.3/KChIP2/DPP6 and K_V_4.3/KChIP2/KCNE2 channels. CHO cells were transiently transfected with cDNAs codifying for different proteins (K_V_4.3/KChIP2, K_V_4.3/KChIP2/DPP6 or K_V_4.3/KChIP2/KCNE2), and the potassium currents were recorded using the whole-cell patch-clamp technique. IQM-266 decreased the maximum peak of K_V_4.3/KChIP2, K_V_4.3/KChIP2/DPP6 and K_V_4.3/KChIP2/KCNE2 currents, slowing their time course of inactivation in a concentration-, voltage-, time- and use-dependent manner. IQM-266 produced an increase in the charge in K_V_4.3/KChIP2 channels that was intensified when DPP6 was present and abolished in the presence of KCNE2. IQM-266 induced an activation unblocking effect during the application of trains of pulses to cells expressing K_V_4.3/KChIP2 and K_V_4.3/KChIP2/KCNE2, but not in K_V_4.3/KChIP2/DPP6 channels. Overall, all these results are consistent with a preferential IQM-266 binding to an active closed state of Kv4.3/KChIP2 and Kv4.3/KChIP2/KCNE2 channels, whereas in the presence of DPP6, IQM-266 binds preferentially to an inactivated state. In conclusion, DPP6 and KCNE2 modify the pharmacological response of K_V_4.3/KChIP2 channels to IQM-266.

## 1. Introduction

Voltage-dependent potassium channels (K_V_4 subfamily) are the main contributors to the cardiac transient outward K^+^ currents (*I*_tof_), having a central role in controlling cardiac excitation and shaping the cardiac action potentials (AP) [1,2,3,4]. There are two *I*_to_ components with distinct recovery kinetics: the fast (*I*_tof_), conducted by K_V_4.2 and K_V_4.3 channels, and the slow (*I*_tos_), conducted by K_V_1.4 channels [5]. *I*_tof_ plays a key role in the early phase of repolarization in many species, including humans [1]. In many pathological conditions such as cardiac hypertrophy, atrial fibrillation or heart failure, a reduction of the *I*_tof_ density and the subsequent prolongation of the action potential duration has been found [6,7,8]. Therefore, the pharmacological *I*_tof_ activation may have therapeutic value in those diseases.

The most comprehensively studied members of the K_V_4 family are K_V_4.2 and the two splice variants of K_V_4.3, K_V_4.3S and K_V_4.3L [9,10]. They present a complementary expression, with K_V_4.2 more abundant in the ventricle and K_V_4.3 in the atria [11,12]. In human heart failure, K_V_4.3L expression rises ∼33% while that of K_V_4.3S falls ∼75% [13].

The α subunits of K_V_4.x channels are enough for the formation of functional K^+^ channels. However, to fully reproduce the *I*_tof_ current, K_V_4 channels need to assemble with other accessory subunits forming *channelosomes*. These protein complexes can be formed by different regulatory (or β) subunits, including KChIPs (potassium channel interacting proteins), DPPs (dipeptidyl-peptidase-like proteins), KCNEs (also termed MinK-related peptides, or MiRPs), KChAPs and K_V_βx subunits [14,15,16,17,18,19].

KChIPs belong to the neuronal calcium sensor superfamily [20]. Within the different KChIP members, KChIP2 is predominantly expressed in the heart [21]. Through its interaction with the amino terminus of the K_V_4.3 α-subunit, KChIP2 induces an increase in the traffic of K_V_4.3 channels to the plasma membrane, a delay in the macroscopic inactivation kinetics, and an acceleration of both the activation and the recovery kinetics from inactivation [14,20]. Interestingly, this general trend is modified by the KChIP-binding ligands. Hence, the knowledge gained from the modulation of the K_V_4.3/KChIP complex by small molecules could open novel therapeutic opportunities for the treatment of cardiovascular diseases in which the *I*_tof_ is reduced [19,22].

DPP6 belongs to the prolyl-oligopeptidase family of serine proteases. It is a single-pass transmembrane protein that can modify channel gating through different mechanisms, which involve direct interactions with α-subunit transmembrane core domains, including the pore and the voltage-sensing domain [17,23,24]. Recent studies have shown that DPP6 exerts its modulation through interactions with the S1 and S2 helices of the K_V_4.2 voltage-sensing domain [25]. DPP6 coexpression with K_V_4.3 accelerates the activation and inactivation kinetics and shifts both activation and inactivation voltage dependencies of K_V_4 channels to more negative potentials [17].

KCNE2, also termed MinK-related peptide 1, or MiRP1, is a single-transmembrane-domain subunit that co-assembles with the K_V_ α subunits, modifying, among other fundamental properties, the channel α subunit composition, trafficking, endocytosis, gating and the effects of regulation by other proteins [16]. MiRP1 slows the rates of K_V_4 activation and inactivation [26] and, during channel recovery from inactivation, it induces an ‘overshoot’ of the current amplitude [16,26], a fact previously detected on the *I*_tof_ from human subepicardial myocytes [27]. Moreover, MiRP1 is thought to interact with the pore domains of the channel and delay both channel activation and inactivation processes [26].

For all the reasons mentioned, the K_V_4-KChIP2-DPP6-KCNE2 *channelosome* could be a promising target in the treatment of cardiac diseases. Moreover, given the fact that IQM-266 has been recently identified as a novel KChIP3 ligand that modulates K_V_4 currents in rat dorsal root ganglion neurons [28], we investigated its binding to KChIP2 and the pharmacological consequences of IQM-266 on the K^+^ currents generated by Kv4.3/KChIP2, Kv4.3/KChIP2/DPP6 and Kv4.3/KChIP2/KCNE2.

## 2. Results

### 2.1. IQM-266 Binding to KChIP2

**Figure 1:**
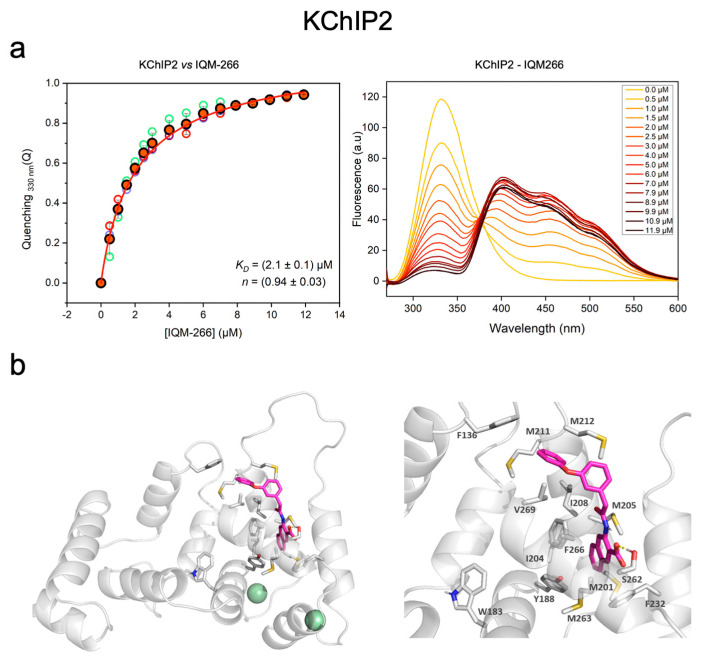
Concentration-dependent interaction between IQM-266 and KChIP2. (**a**) Interaction between IQM-266 and KChIP2. Left panel shows the plot of the quenching of tryptophan by FRET towards IQM-266. Data from the five repetitions (open symbols) are shown as well as the average value (red-filled symbols) and error bars as s.d. The red line represents the fit to a Hill equation. Right panel shows the fluorescence emission spectra (λ_ex_ = 260 nm) from a representative titration of KChIP2 (5 µM) with increasing concentrations of IQM-266. (**b**) Representation of IQM266 binding pose with the homology model of KChIP2. Residues within the binding pocket, together with Trp, are indicated. For clarity, only polar hydrogens are included and the hydrogen bond is depicted as a discontinuous yellow line (PyMOL Molecular Graphics System, Version 2.0 Schrödinger, LLC. New York, NY, USA).

We first tested the interaction between IQM-266 (3-(2-(3-phenoxyphenyl)acetamido)-2-naphthoic acid) and KChIP2. IQM-266 binding was monitored by quenching of the intrinsic tryptophan fluorescence emission of KChIP2 (Figure 1a) caused by fluorescence resonance energy transfer (FRET) towards the IQM-266 chromophore. The fact that there are tryptophan residues in close proximity to the binding site and that the spectral overlap between the two chromophores is suitable allows an efficient FRET process. Using the spectral features of the emission of KChIP2 and the absorption of IQM-266, the estimated quantum yield of the tryptophan for the parent KChIP3, and the molar absorptivity of IQM-266, we estimated a Förster distance, R_0_, of 20.4 Å (assuming free rotation of the dyes, which is likely not a totally valid assumption but provides a rough estimation of the distance) [29]. FRET is evident by the quenching in the tryptophan fluorescence and the concomitant increase in the emission of IQM-266 (Figure 1a), which indicates close proximity between the chromophore of IQM-266 and the KChIP2 tryptophan. We estimated the amount of quenching to the tryptophan emission being at saturating concentrations of IQM-266 around 0.92. This quenching efficiency corresponded to an apparent distance tryptophan–IQM-266 of circa. 13.5 Å. Next, to determine IQM-266 affinity, the quenching of tryptophan emission at 330 nm was plotted versus the concentration of IQM-266 (Figure 1a) and the dissociation constant was calculated using a Hill equation (see Methodology). Our experiment showed that IQM-266 binds to KChIP2 with a dissociation constant of 1.9 ± 0.1 µM and a Hill coefficient of around 1 (Figure 1a).

To gain further insights into the binding pocket of IQM-266, molecular docking studies were carried out. As there is no known 3D structure of KChIP2, homology models were built on the base of the NMR structure of KChIP3 (PDB ID 2JUL, 15 structures) [30], sequence identity of 77% with hKChIP2 C-terminal region. Five homology models were generated using the Schrödinger’s Prime module. Additionally, a model was built on the base of the X-ray structure of KChIP1 (72% identity) in complex with the Kv4.3 assembly domain T1 (PDB ID 2I2R) [31]. In this structure the N-terminal helix of the channel is accommodated in a KChIP1 cleft and, thus, there is a reordering of the protein, as particularly KChIP1 H10 moves from its position in 2JUL. These models provided several protein conformations for the docking studies. Then, IQM-266 was docked in a site centered on Tyr188, Ile208 and Phe232. IFD studies identified several locations and poses of the ligand within KChIP2; as expected, a wider variety was observed for the model generated based on 2I2R due to the large pocket size. Based on the score and visual inspection, a pose was selected (Figure 1b). The IFD studies indicated that IQM-266 is located in a cleft surrounded by KChIP2 EF-hands 3 and 4 and helix H10. In this pose, the IQM-266 carboxylic acid is participating in a hydrogen bond with Ser262 from KChIP2 H10. The naphthyl ring is surrounded by hydrophobic residues, namely Met201, Ile204, Met263, Tyr188, Phe266, and a T-interaction is observed with Phe232. Similarly, the Ph-O-Ph moiety is accommodated in a hydrophobic cavity flanked by Phe136, Ile208, Met211, Met212 and Val269. IQM-266 is at 12 Å from Trp183, in agreement with the apparent distance tryptophan–IQM-266 obtained from the experimental data.

### 2.2. Effects of IQM-266 on K_V_4.3/KChIP2, K_V_4.3/KChIP2/DPP6 and K_V_4.3/KChIP2/KCNE2 Channels

Human cardiac *I*_tof_ is generated by the activation of K_V_4.3 channels assembled with regulatory subunits, from which the most important ones are KChIP2, DPP6 and KCNE2 [16,17,24,32]. Considering that IQM-266 is a KChIP2 ligand, we analyzed the effects of IQM-266 in CHO cells transfected with K_V_4.3 channels and KChIP2 together with DPP6 or KCNE2.

**Figure 2:**
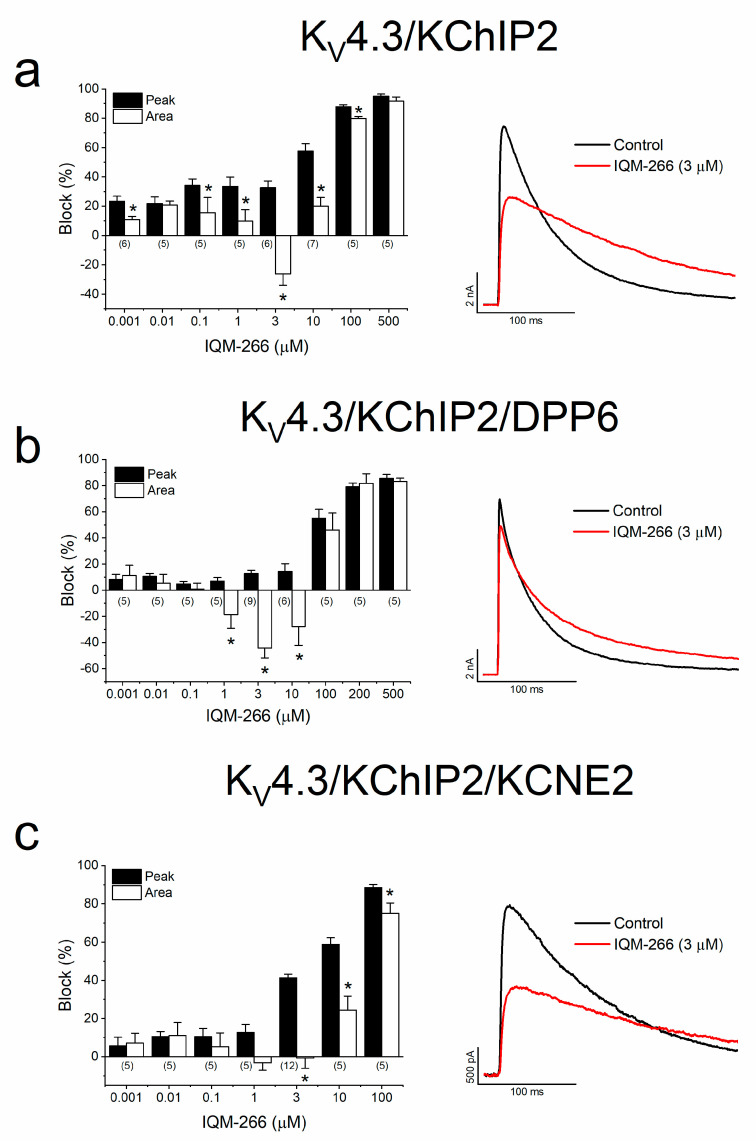
Concentration-dependent interaction between IQM-266 K_V_4.3/KChIP2, K_V_4.3/KChIP2/DPP6 and K_V_4.3/KChIP2/KCNE2. (**a**–**c**) Inhibition or increase of the current induced by IQM-266 in K_V_4.3/KChIP2 (**a**), K_V_4.3/KChIP2/DPP6 (**b**) and K_V_4.3/KChIP2/KCNE2 (**c**) channels measured at the maximum peak current and in the charge (measured as the area of the current during the application of a 250 ms pulse to +60 mV). Right panels show current records obtained in the absence and in the presence of IQM-266 (3 µM). Each bar represents the mean ± S.E.M. of the number of cells shown in the bars. Statics was performed by a paired Student *t*-test comparing the block produced at the peak current and the block/increase produced at the charge for each concentration of IQM-266 tested. *: *p* < 0.05.

First, we analyzed the effects of different concentrations of IQM-266 (between 0.001 and 500 μΜ) on K_V_4.3/KChIP2, K_V_4.3/KChIP2/DPP6 and K_V_4.3/KChIP2/KCNE2 channels expressed in CHO cells (Figure 2).

The effects were measured at the maximum peak current and on the charge (measured as the area underneath the current). In K_V_4.3/KChIP2 channels, a concentration-independent block of the peak current between 0.001 and 1 μΜ was observed, showing slighter effects on the charge (Figure 2a). Previously, we have demonstrated that IQM-266 (3 μM) produced an activation of K_V_4.3/KChIP3 [28] in the charge during the application of 250 ms depolarizing pulses from −80 to +60 mV. This was also observed in K_V_4.3/KChIP2, but to a greater extent. This effect is the result of the equilibrium between two effects: (i) block produced on the maximum peak current and (ii) a slowing effect on the inactivation kinetics. At higher concentrations, this compound produced a concentration-dependent decrease both at the maximum peak current (Supplementary Figure S1 with an EC_50_ of 13 μM) and at the charge.

In the presence of DPP6 (K_V_4.3/KChIP2/DPP6) (Figure 2b), the IQM-266-induced block of the maximum peak current was almost negligible at concentrations lower than 100 μΜ. However, under these conditions, the activating effect produced by IQM-266 in K_V_4.3/KChIP2/DPP6 channels was significantly greater and appeared in a wider range of concentrations (1–10 μΜ) than in K_V_4.3/KChIP2 channels. These effects may be the consequence of a slight block of the maximum peak current together with a slower inactivation kinetics. Indeed, IQM-266 blocked the maximum peak current generated by K_V_4.3/KChIP2/DPP6 channels exhibiting an EC_50_ of 80 μΜ (Supplementary Figure S1). However, lower concentrations were needed to increase the time constant of inactivation.

Finally, in the presence of KCNE2 (K_V_4.3/KChIP2/KCNE2), the activator effect produced by IQM-266 on K_V_4.3/KChIP2 channels was abolished (Figure 2c). However, and similarly to that observed in K_V_4.3/KChIP2 channels, IQM-266 slowed down the inactivation of the current. However, in contrast to that observed in K_V_4.3/KChIP2 channels, the block produced by this compound, measured at the maximum peak current, was greater (Supplementary Figure S1; with an EC_50_ of 5 μM) and the slowing of the inactivation of the current was lesser.

In order to characterize the increase of the current induced by IQM-266, we studied the effects of this compound at a concentration of 3 μM on K_V_4.3/KChIP2, K_V_4.3/KChIP2/DPP6 and K_V_4.3/KChIP2/KCNE2 channels.

### 2.3. Time-Dependent Effects of IQM-266 on K_V_4.3/KChIP2, K_V_4.3/KChIP2/DPP6 and K_V_4.3/KChIP2/KCNE2 Channels

**Figure 3:**
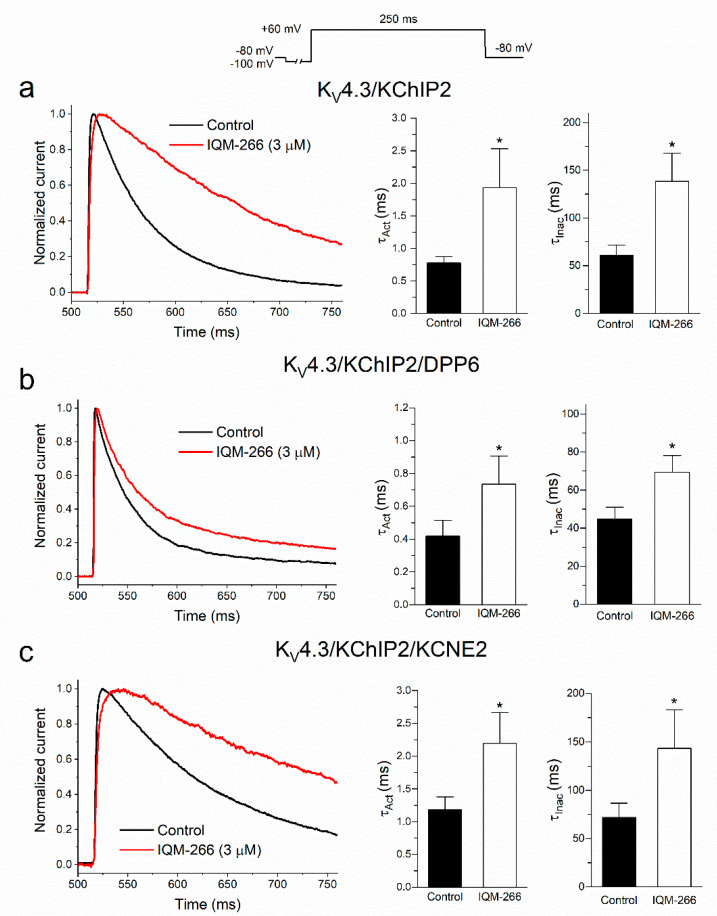
Activation and inactivation kinetics of K_V_4.3/KChIP2 (**a**), K_V_4.3/KChIP2/DPP6 (**b**) and K_V_4.3/KChIP2/KCNE2 (**c**) currents in the absence and in the presence of IQM-266 (3 µM). Left panels show normalized current records under the three experimental conditions. These current records were fitted to a monoexponential equation in order to obtain the τ_Act_ values. Middle panels show the histogram representing the τ_Act_ under the different experimental conditions. Right panels show the histograms representing the τ_Inac_ values under the three experimental conditions. Each bar represents the mean ± S.E.M. of *n* = 8 cells (from *n* = 5 transfections) for K_V_4.3/KChIP2 channels, *n* = 8 (from *n* = 6 transfections) for K_V_4.3/KChIP2/DPP6 channels and *n* = 10 (from *n* = 9 transfections) for K_V_4.3/KChIP2/KCNE2 channels. *: *p* < 0.05.

The effects on the activation kinetics produced by IQM-266 were assessed by fitting the current traces of K_V_4.3/KChIP2, K_V_4.3/KChIP2/DPP6 and K_V_4.3/KChIP2/KCNE2 to a monoexponential process, with the activation time constant (τ_Act_), both in the absence and in the presence of IQM-266 (3 μM) (Figure 3).

Similarly, the inactivation kinetics (τ_Inac_) were analyzed by fitting the inactivation process. As it can be observed in Figure 3, the time constant of both the activation and inactivation processes were significantly increased in the presence of IQM-266 under the three experimental conditions. The slowing of the activation and inactivation kinetics produced by this compound was greater on K_V_4.3/KChIP2 (Figure 3a), followed by K_V_4.3/KChIP2/KCNE2 (Figure 3c) and finally by K_V_4.3/KChIP2/DPP6 (Figure 3b) currents.

### 2.4. Voltage Dependence of the Block Produced by IQM-266 on K_V_4.3/KChIP2, K_V_4.3/KChIP2/DPP6 and K_V_4.3/KChIP2/KCNE2 Channels

**Figure 4:**
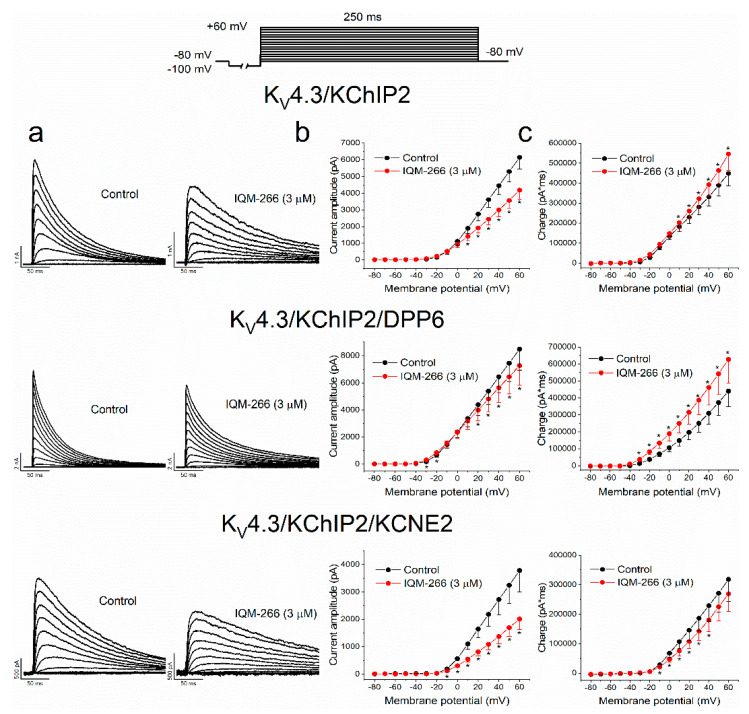
Voltage dependence interaction between IQM-266 and K_V_4.3/KChIP2 K_V_4.3/KChIP2/DPP6 and K_V_4.3/KChIP2/KCNE2 channels. (**a**) Original recordings obtained elicited by the activation of K_V_4.3/KChIP2, K_V_4.3/KChIP2/DPP6 and K_V_4.3/KChIP2/KCNE2 channels in the absence and in the presence of IQM-266 (3 µM) after applying the pulse protocols shown in the upper part of the figure. (**b**) I-V relationship of the currents generated by K_V_4.3/KChIP2 (*n* = 7 cells from *n* = 7 transfections), K_V_4.3/KChIP2/DPP6 (*n* = 8 cells from *n* = 6 transfections) and K_V_4.3/KChIP2/KCNE2 (*n* = 10 cells from *n* = 8 transfections) channels in the absence and in the presence of IQM-266 at 3 μM. (**c**) Q-V relationship of the charge through K_V_4.3/KChIP2 (*n* = 6 cells from *n* = 5 transfections), K_V_4.3/KChIP2/DPP6 (*n* = 8 cells from *n* = 6 transfections) and K_V_4.3/KChIP2/KCNE2 (*n* = 10 from *n* = 8 transfections) channels in the absence and in the presence of IQM-266 at 3 μM, measured at the area under the current during the application of depolarizing pulse protocol shown in the upper part of the figure. Each point represents the mean ± S.E.M. *: *p* < 0.05.

Figure 4a shows superimposed traces obtained after applying the pulse protocol represented in the top of the Figure, in the absence and in the presence of IQM-266 (3 μM).

As previously stated, IQM-266 decreased the maximum peak current and slowed down the inactivation kinetics under the three experimental conditions. By plotting the maximum peak current at each membrane potential tested for both control and after perfusion of the cells with IQM-266, the I-V relationships were obtained (Figure 4b). As it is shown, the maximum peak current block, produced by IQM-266 under the three experimental conditions, was greater in K_V_4.3/KChIP2/KCNE2 channels, followed by K_V_4.3/KChIP2 and K_V_4.3/KChIP2/DPP6. Under the three experimental conditions, maximum peak block sharply increased in the range of voltage membrane activation. Figure 4c shows the charge–voltage (Q-V) relationship obtained after measuring the charge (measured as the area per unit time) in the absence and in the presence of IQM-266 (3 μM). We observed that IQM-266 produced an increase in the charge in K_V_4.3/KChIP2 channels that was intensified when DPP6 was present and abolished in the presence of KCNE2. From the I-V relationships, we obtained the activation curves. Their V_h_ and the s parameters were not modified in K_V_4.3/KChIP2 and K_V_4.3/KChIP2/KCNE2 currents, whereas we observed a negative shift of the V_h_ induced by IQM-266 (3 μM) in the activation curve generated by K_V_4.3/KChIP2/DPP6 channels (Supplementary Figure S2).

### 2.5. Use-Dependent Effects of IQM-266 on K_V_4.3/KChIP2, K_V_4.3/KChIP2/DPP6 and K_V_4.3/KChIP2/KCNE2 Channels

**Figure 5:**
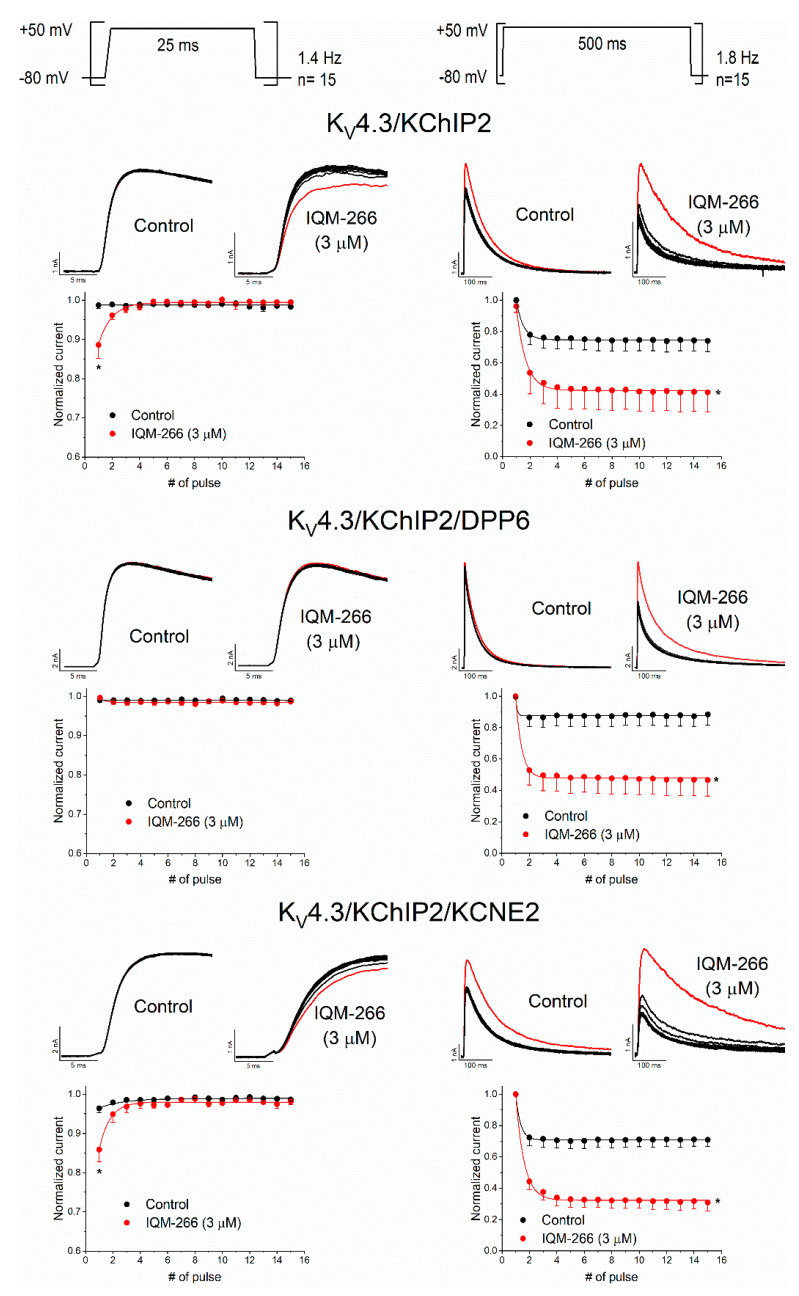
Use-dependent effects produced by IQM-266 on K_V_4.3/KChIP2, K_V_4.3/KChIP2/DPP6 and K_V_4.3/KChIP2/KCNE2 channels after applying the two pulse protocols shown in the top of the figure. In the three panels, the top one shows current records obtained after applying the pulse protocols shown in the upper part of the figure. The red current represents the first current record of each train. Below the current records, the normalized current versus the first pulse is shown. Each point represents the mean ± S.E.M. of *n* = 5 cells (from *n* = 5 transfections) for K_V_4.3/KChIP2 channels, *n* = 6 cells (from *n* = 5 transfections) for and K_V_4.3/KChIP2/KCNE2 channels and *n* = 6 cells (from *n* = 5 transfections) of K_V_4.3/KChIP2/DPP6 channels. *: *p* < 0.05.

Although IQM-266 (3 μM) produced an increase in the current of K_V_4.3/KChIP2 and K_V_4.3/KChIP2/DPP6 channels, this effect may not be attained during a single action potential. Therefore, we tested whether IQM-266-induced effects displayed use dependence by applying two different pulse protocols, which may lead us to discriminate between the channel state for which IQM-266 has a greater affinity. In both protocols, 15 pulses from −80 mV to +50 mV of 25 ms (short-pulse train) or 500 ms (long-pulse train) at frequencies of 1.4 and 1.8 Hz, respectively, were applied (Figure 5). During the short-pulse train, channels shift from the closed to the open state without inactivate, whereas during the long-pulse train, K_V_4 channels passage from the closed to the open and, finally, to the inactivated state.

After applying a short-pulse train under control conditions, the magnitude of the current K_V_4.3/KChIP2 was not modified. However, in the presence of IQM-266 (3 μM), the magnitude of the current elicited during the first pulse of the train exhibited the smallest amplitude and the magnitude of the current after applying the successive pulses exponentially increased until a saturated value was obtained. Similar results were obtained in K_V_4.3/KChIP2/KCNE2 channels. This increase in the current during the short-pulse train application may be explained by an activation unblocking process, as it has been reported in sodium channels with other drugs [33,34]. Interestingly, this increase in the magnitude of the current during the application of a short-pulse train was not observed in K_V_4.3/KChIP2/DPP6 channels.

After the application of the long-pulse train protocol to K_V_4.3/KChIP2 channels in the absence of IQM-266, a decrease of a 26.0 ± 6.8% (*n* = 5) without changes in the inactivation kinetics of the current was observed, which is the consequence of the accumulation of inactivation. After perfusion with IQM-266, this decrease of the current during the long-pulse train was enhanced to 54.6 ± 16.4% (*n* = 5, *p* < 0.05). These results can be explained if IQM-266 also binds to the inactivated state of the channel. Moreover, during the application of the long-pulse train, the kinetics of the first pulse of the train exhibited similar monoexponential kinetics than under steady state conditions (similar to that observed in the I-V relationship), whereas the inactivation kinetics of the current became slightly faster after applying successive pulses. This small acceleration of the inactivation kinetics of the current during the application of the long-pulse train can represent the binding time constant of IQM-266 to the inactivated state of the K_V_4.3/KChIP2 channel.

Similar results were observed when KCNE2 was present, in which the decrease of the current during the train was also greater in the presence than in the absence of IQM-266 (27.9 ± 4.9% vs. 67.8 ± 6.3% in the absence and in the presence of IQM-266, respectively, *n* = 6, *p* < 0.05). Furthermore, the kinetics of the current elicited after applying the first pulse of the train were slightly slower than the currents generated after the successive currents of the train pulses. Likewise, in the presence of DPP6, a greater decrease in the magnitude of the current was observed when the long-pulse train protocol was applied (4.6 ± 2.9% vs. 48.1 ± 10.7% in the absence and in the presence of IQM-266 respectively, *n* = 5; *p* < 0.05). However, under these conditions, the kinetics of the current were not changed during the application of the long-pulse train. The use-dependent effects observed during the application of the long-pulse train protocols could be explained if the recovery process in the presence of IQM-266 is slower.

**Figure 6:**
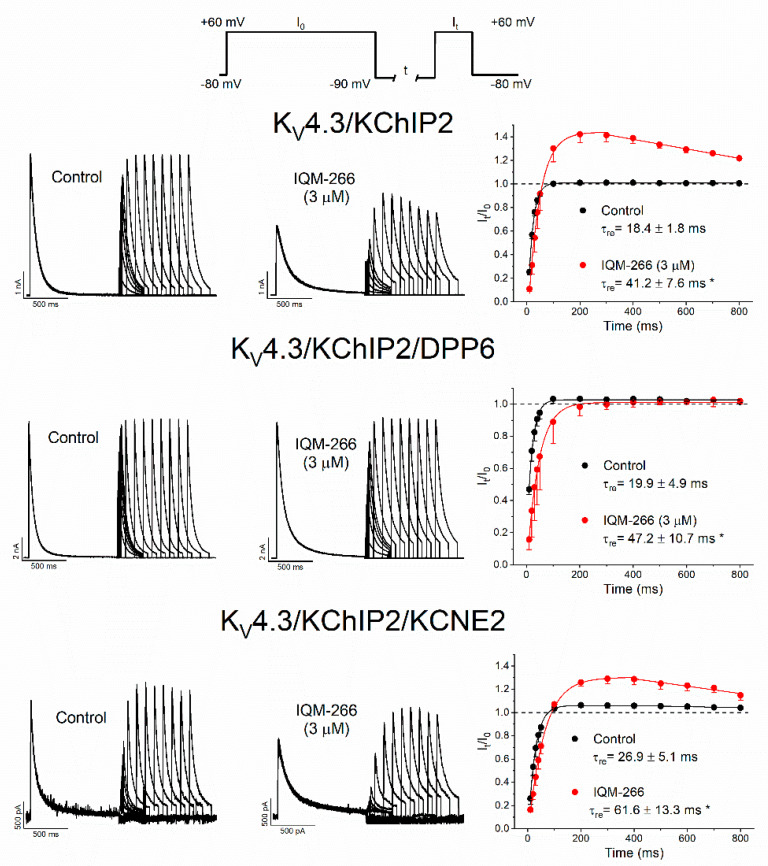
Effects of IQM-266 on the recovery kinetics from inactivation of K_V_4.3/KChIP2, K_V_4.3/KChIP2/DPP6 and K_V_4.3/KChIP2/KCNE2 channels. Left panel shows original records obtained after applying the pulse protocol shown in the upper panel of the figure. Right panels show the data obtained after plotting the I_t_/I_0_ vs. the interpulse period separating both the conditioning and the test pulse. Each point represents the mean ± S.E.M. of *n* = 5 cells (from *n* = 5 transfections) for K_V_4.3/KChIP2, *n* = 6 cells (from *n* = 5 transfections) for K_V_4.3/KChIP2/DPP6 channels and of *n* = 6 cells (from *n* = 5 transfections) for K_V_4.3/KChIP2/KCNE2 channels. *: *p* < 0.05.

In order to test this hypothesis, a double pulse protocol from −80 mV to +60 mV was applied, consisting of a 1 s conditioning (I_0_), followed by a 250 ms test pulse (I_t_) to +60 mV, applied after a variable time period at −90 mV. This protocol was applied before and after perfusion with IQM-266 (3 μM). Figure 6 shows original records obtained after applying the pulse protocol shown in the top of Figure 6 in cells transfected with K_V_4.3/KChIP2 in control and in the presence of IQM-266. The ratio I_t_/I_0_ was plotted versus the time recovery interval. These data were fitted to a monoexponential equation from which the recovery time constant (τ_re_) was calculated.

Under the three experimental conditions, IQM-266 slowed the τ_re_, which is consistent with the results obtained after applying long-pulse trains (Figure 5). However, in K_V_4.3/KChIP2 and in K_V_4.3/KChIP2/KCNE2 channels, an overshoot between 75 and 220 ms was observed and afterwards it decreased. These results are consistent with the activation unblocking process observed after applying the short train pulses. On the other hand, in cells transfected with K_V_4.3/KChIP2/DPP6 channels, the recovery process was also slower after perfusion with IQM-266, but the overshoot was not observed, which agrees with the lack of activation unblocking process observed during the application of short train pulses.

## 3. Discussion

In the present article we have identified IQM-266 as a new ligand of KChIP2 and studied the pharmacological consequences of the assembly of three regulatory subunits (KChIP2, DPP6 and KCNE2) on K_V_4.3 channels in the presence of IQM-266. In this study we show that: (1) IQM-266 is a KChIP2 ligand, (2) the increased effect on the charge observed in K_V_4.3/KChIP2 channels is enhanced in the presence of DPP6 and abolished with KCNE2; (3) block of the maximum peak current of K_V_4.3/KChIP2, K_V_4.3/KChIP2/DPP6 and K_V_4.3/KChIP2/KCNE2 channels is concentration-, voltage-, time- and use-dependent; (4) during the application of trains of pulses to cells expressing K_V_4.3/KChIP2 and K_V_4.3/KChIP2/KCNE2, but not in K_V_4.3/KChIP2/DPP6 channels, an activation unblocking effect was observed. All these results are consistent with a preferential IQM-266 binding to an active closed state of Kv4.3/KChIP2 and Kv4.3/KChIP2/KCNE2 channels, whereas in the presence of DPP6, IQM-266 binds preferentially to an inactivated state.

During the last decade, a lot of effort has been made in developing new small molecules able to bind accessory subunits, such as KChIP2, and which are capable of increasing K_V_4 channel function [19,28,35,36,37]. Until now, there have been only two compounds capable of increasing the K_V_4.3/KChIP2 current (NS5806 and NS3623) [19]. Both exhibit a dual effect, inhibiting or enhancing the K_V_4 current under different experimental conditions or in different cell types [36]. In fact, the presence or the absence of certain β subunits led to a greater or to a lesser extent of their activator effect on the current. Here, we identified IQM-266 as a new KChIP2 ligand able to increase the K_V_4.3/KChIP2 current.

The current inhibition of the maximum peak current produced by IQM-266 was much greater in K_V_4.3/KChIP2/KCNE2 and K_V_4.3/KChIP2 than in K_V_4.3/KChIP2/DPP6 channels (IC_50_ values of 5, 13 and 80 μM, respectively). Moreover, IQM-266 also slowed to different extents the inactivation time course of these channels. The equilibrium between these two effects will determine the activator effect observed on the charge at certain concentrations. Therefore, in those conditions in which the blocking effect produced by IQM-266 of the maximum peak current are very high, the activator effect will not be observed (K_V_4.3 and K_V_4.3/KChIP2/KCNE2), whereas if the block of the maximum peak current is much lower, the activator effect will be more evident (K_V_4.3/KChIP2/DPP6). IQM-266 produced an intermediate effect on K_V_4.3/KChIP2 channels than those produced in K_V_4.3 and K_V_4.3/KChIP2/DPP6 channels and, thus, we observed an increase of the charge at 3 μM, but not at other ranges of concentrations, such as in K_V_4.3/KChIP2/DPP6 channels.

It is known that KChIP2 interacts with the N-terminus of the channel and DPP6 with the voltage sensor, specifically with the S1 and S2 transmembrane segments [25,31]. Several studies point out that KCNE2 interacts with the pore of the channel, although there are not structural studies confirming this issue [38]. Recently, we reported that IQM-266 decreased the maximum peak of K_V_4.3 current, without increasing the charge [28]. In our experiments, we analyzed the interaction between IQM-266 and KChIP2 (K_D_ value of 2 μM), K_V_4.3/KChIP2, K_V_4.3/KChIP2/DPP6 and K_V_4.3/KChIP2/KCNE2 channels. In these three channels, the N-terminus of K_V_4.3 is interacting with KChIP2. In K_V_4.3/KChIP2/DPP6 channels, the transmembrane segment of DPP6 interacts with the voltage sensor of the channel, which can explain the shift of the activation curve produced by IQM-266 (Supplementary Figure S2). Furthermore, it is interesting to note that IQM-266 displaces the activation curve only in the presence of DPP6, but not in the presence of KChIP2 or KCNE2, which have been described to interact with the N-terminus and the pore, respectively [25,38]. Altogether, our findings point out that DPP6 is an essential auxiliary subunit for the observed voltage shift. Additionally, the IQM-266-increased activator effects observed in the presence of DPP6 could be explained because KChIP2 and DPP6 produce additive effects on the K_V_4.3 channel since they interact with different domains, so the differential effects of IQM-266 on these conditions would also be additive. Finally, in K_V_4.3/KChIP2/KCNE2 channels the activating effect of IQM-266 observed in the absence of KCNE2 was abolished, which may be attributed to greater IQM-266 inhibitory effects of the peak current elicited by K_V_4.3/KChIP2/KCNE2 channels.

The results obtained in the present study on K_V_4.3/KChIP2 and K_V_4.3/KChIP2/KCNE2 channels may be explained by an interaction of IQM-266 with the closed-activated state of these channels and with a very low affinity for the open channel state of these two channels. These conclusions are based on: (i) the activation unblocking effects observed during the application of short train pulses similar to those reported in sodium channels and other drugs [33,34], and the overshoot of the recovery kinetics from inactivation, and (ii) the increase in the block of the maximum peak current observed in the range of voltage membrane activation of these channels. On the other hand, in K_V_4.3/KChIP2/DPP6 channels, IQM-266 did not produce any effect after the application of short train pulses, which may suggest, together with the lack of overshoot in the recovery kinetics from inactivation, that this compound does not exhibit a high affinity for the active closed state of these channels, although we cannot rule out a very fast interaction between IQM-266 and K_V_4.3/KChIP2/DPP6 channels, in such a way that we cannot observe in the current records. Furthermore, IQM-266 seems to interact with the inactivated state of K_V_4.3/KChIP2, K_V_4.3/KChIP2/DPP6 and K_V_4.3/KChIP2/KCNE2 channels, which is consistent with the use-dependent effects observed after applying long-pulse trains and also with the slowing produced on the recovery kinetics of inactivation.

Future studies on the K_V_4.3 *channelosome* are needed to identify new interactors and their physiological and pathological roles and, thus, in this way, we could find out new therapeutic targets for cardiac diseases involving *I*_tof_. In conclusion, DPP6 and KCNE2 modify the pharmacological response of K_V_4.3/KChIP2 channels to IQM-266. The study of these and other new ligands may open opportunities for drug development.

## 4. Materials and Methods

All experiments shown in the present study were performed following European Parliament 2010/63/EU and the rules of the Helsinki Declaration. All experimental procedures followed the guidelines for ethical care of the European Union (2012/63/EU).

### 4.1. Cellular Cultures and Transient Transfection

The experiments shown in the present study were performed in CHO-K1 cells (*Chinese Hamster Ovary*, CHO) from the American Type Culture Collection (Rockville, MD, USA). Cells were cultured in Iscove’s modified Eagle’s medium supplemented with 10% (*v*/*v*) fetal bovine serum (FBS), 1% (*v*/*v*) L-Glutamine and antibiotics (100 IU/mL penicillin and 100 mg/mL streptomycin), all from Gibco, Paisley, UK, at 37 °C in a 5% CO_2_ atmosphere.

We used K_V_4.3S (2 μg, cloned into pBK-CMV (given by Dr. D.J. Snyders, University of Antwerpen, Belgium)) and KChIP2c (given by Dr. R. Bähring, University of Hamburg) (1 μg, cloned into IRES mCherry), KCNE2 (2 μg) and DPP6S (2 μg), both cloned into the pIRES2-EGFP vectors (kindly provided by Dr. S. Kaemmerer). Kv4.3S was chosen because Kv4.3S-KChIP channels exhibited up to 8-fold greater current augmentation, 40% slower inactivation and 5 mV-shifted steady-state inactivation compared to Kv4.3L-KChIP2 [39]. Since we are studying the effects of a compound that targets KChIP2, we choose Kv4.3S in order to better distinguish its effects on this auxiliary beta subunit. All these cDNAs were transfected using Fugene-6 (Promega, Madison, WI, USA) following manufacturer’s instructions. Then, 48 h post-transfection, cells were removed from culture plates using TrypLE^TM^ Express (Thermo Fisher Scientific Inc., Waltham, MA, USA). Since the level of expression of KChIP2 may be very important for the drug effects, only cells cotransfected with K_V_4.3 and KChIP2 that exhibit a recovery kinetics from inactivation between 14 and 24 ms were selected for electrophysiological recording.

### 4.2. Purification Protocol

KChIP2 protein was cloned, expressed and purified as follows: a PCR fragment coding for the open reading frame from residue His88 to the C-term of KChIP2, flanked by NheI/BlpI restriction sites at the 5′ and 3′ ends, respectively, was cloned into a His-tagged pET28a vector (GenScript, Piscataway, NJ, USA). This plasmid was transformed into *Escherichia coli* BL21 Star(DE3) cells (Thermo Fisher Scientific Inc., Waltham, MA, USA). KChIP2 was over-expressed overnight at 289K after induction with 0.3 mM isopropyl B-D-1-thiogalactopyranoside.

Cultures were harvested by centrifugation and resuspended in buffer A (20 mM Tris pH 7.5; 500 mM NaCl, 10 mM imidazole). Cells were lysed by sonication, centrifuged at 16,000 rpm in an SS-34 rotor, and the supernatant was loaded onto a Ni^2+^ affinity column (GE Healthcare, Chicago, IL, USA) previously equilibrated in buffer A. The column was washed with 10× CVs of buffer B (20 mM Tris pH 7.5, 500 mM NaCl, 40 mM imidazole). Protein was eluted with buffer C (20 mM Tris pH 7.5, 500 mM NaCl, 500 mM imidazole). Then, protein buffer was changed to buffer D (20 mM Tris pH 7.5, 100 mM NaCl) for TEV cleavage of the His-tag overnight at 4 °C under gentle shaking. KChIP2 was again loaded on a Ni^2+^ affinity column to remove TEV contamination. Protein purity and homogeneity was confirmed by SDS-PAGE and gel filtration chromatography.

### 4.3. Concentration Determination

Protein determination was measured using a NanoDrop 2000c spectrophotometer (Thermo Fisher Scientific Inc., Waltham, MA, USA) monitoring the absorbance at 280 nm, using a molar absorptivity coefficient of 15,930 M^−1^ cm^−1^, estimated through the protein sequence.

### 4.4. Chemistry

(3-(2-(3-Phenoxyphenyl)acetamido)-2-naphthoic acid (IQM-266) was synthetized in the Instituto de Química Médica (IQM, CSIC) following the previously described protocol [28].

### 4.5. Fluorescence Binding Assay

Fluorescence binding assay was carried out at 25 °C on a Jasco FP-8500 spectrofluorometer (JASCO Deutschland GmbH, Pfungstadt, Germany). Different IQM-266 concentrations were added to KChIP2 (5 µM) in 50 mM HEPES pH = 7.4, 100 mM NaCl and 1 mM CaCl_2_ at room temperature. Fluorescence emission was scanned from 270 nm to 600 nm (1 s integration time) with an excitation wavelength of 260 nm and slit widths of 2.5 nm for excitation and emission. All the spectra were corrected for background fluorescence by subtracting a blank scan of the solvent solution. Fluorescence resonance energy transfer (FRET) between KChIP2 and IQM-266 was monitored by the decrease of the intrinsic tryptophan fluorescence emission at 330 nm and analyzed using the equation:

Quenching = 1 − (*F_c_*/*F*_0_)

where *F_c_* and *F*_0_ are the fluorescence emission at 330 nm in the presence and in the absence of IQM-266, respectively.

Quenching at different IQM-266 concentrations was analyzed by a Hill equation:

Q = Q_max_ × ([IQM-266]/(KD^n^ + [IQM-266]^n^)

where Q_max_ represents the calculated maximal quenching, [IQM-266] the IQM-266 concentration, K_D_ the equilibrium dissociation constant and n is the Hill coefficient, using Origin 2020 software (Origin Labs Inc., Northampton, MA, USA). Five complete repeats were carried out and error bars are reported as the standard deviations (s.d.).

### 4.6. Homology Modeling

Homology models were built for human KChIP2 (hKChIP2) isoform 2, the one located in heart. Five models were generated on the base of the NMR structure of the *Mus musculus* KChIP3 (pdb code 1JUL, 15 structures) and one on the base of the X-ray structure of KChIP1 (PDB ID 2I2R) [30,31]. The template structures were prepared using the Protein Preparation Wizard tool of the Schrödinger Suite of Programs [40,41] water molecules beyond 5 Å from the protein were deleted. For homology modelling we used the Prime application in Schrödinger Suite 2019 [41,42,43]. Energy minimization was done by using OPLS3e force field and refinement was carried out until average mean square deviation of the non-hydrogen atoms reached 0.3 Å. PROCHECK [44,45] was used to assess the quality of the models. Six models were generated, five using as protein target 2JUL (models 1, 3, 4, 7 and 12) and one 2I2R. These models represent different conformations of the protein, which is of interest for the subsequent docking studies.

### 4.7. Ligand Preparation

IQM266 was constructed using Maestro 2019-4 (Maestro, Schrödinger, LLC, New York, NY, USA, 2019) and prepared using the LigPrep application in the Schrödinger Suite 2019-4 [46], which optimizes ligand structures, corrects improper bond distances and bond orders, generates ionization states and performs energy minimization.

### 4.8. Docking Studies

The docking site was defined as a cubic box of 10 Å centered on Tyr188, Ile208 and Phe232, based on the previous identified pocket for the KChIP2 and KChIP3 activator, NS5806, in its binding to KChIP3 [29]. The IFD protocol 2019-4 [46,47,48,49,50] within the Schrödinger Suite was used for these studies. IFD allows sidechain flexibility for residues around 5 Å from the ligand. Up to 20 poses for ligands were collected. In this protocol, for the initial Glide docking, to reduce steric clashes, the van der Waals radii (rdW) of both protein and ligand were scaled to 0.5, and the ligands were docked into the fixed KChIP3 protein. Then, to optimize the side chains of the residues within 5 Å of the ligand, Prime was used. Finally, the ligands were re-docked into these new receptor conformations using Glide and the default rdW radii. The poses were ranked using IFD score. Selection was based on the best-ranked poses and on visual inspection.

### 4.9. Electrophysiology Methods

Before performing patch-clamp experiments, fluorescent cells (positive to mCherry and/or GFP) were isolated using Fluorescence Activated Cell Sorting (FACS) procedure and preserved in DMEM until being used. A suspension of cells was placed on a chamber mounted on the stage of an inverted microscope.

Potassium currents elicited after the activation of CHO transfected cells were recorded using the whole-cell configuration of the patch-clamp technique with an Axopatch-200B amplifier (Molecular Devices Co., San Jose, CA, USA) and were low-pass filtered and sampled at 2 kHz with an analogue to digital converter (Digidata 1322A, Molecular Devices Co., San Jose, CA, USA). Experiments were performed at room temperature (22 ± 2 °C).

Borosilicate glass capillaries (GD-1, Narishige, London, UK) were pulled with a programmable horizontal puller (P-87, Sutter Instruments, Novato, CA, USA) and subsequently they were polished with a microforge (MF-83, Narishige, London, UK). After heat-polishing, the average pipette tip resistance, previously filled with the internal solution, ranged between 2.5 and 3.5 MΩ. Gigaohm seal formation was achieved by suction (2–5 GΩ). After seal formation, the cell was lifted from the bath and the membrane patch was ruptured with a brief additional suction. Capacitance and series resistance compensation were optimized and usually 80% of compensation of the effective access resistance was accomplished. The obtained data were stored in a personal computer and were analyzed using the clampfit 11.1 utility of pClamp 11.1 software (Molecular Devices Co., San Jose, CA, USA). In addition, Origin 2019 (Origin-Lab Co., Northampton, MA, USA) and clampfit 11.1 were used to perform least squares fitting as well as data presentation. The electrode solution contained (in mM): 80 K-Aspartate, 42 KCl, 3 phosphocreatine, 10 KH_2_PO_4_, 3 Mg-ATP, 5 HEPES-K, 5 EGTA-K (adjusted to pH = 7.25 with KOH). Cells were continuously perfused with an external solution composed by (in mM): 145 NaCl, 4 KCl, 1.8 CaCl_2_, 1 MgCl_2_, 10 HEPES-Na and 10 glucose (adjusted to pH = 7.4 with NaOH) [51,52]. All experiments were repeated at least five times on separate cells.

K_V_4.3/KChIP2 (with DPP6 or KCNE2) currents were evoked following the application of 250 ms depolarizing pulses from −80 to +60 mV in 10-mV increments to obtain the current–voltage relationship (I-V). After control data were obtained, bath perfusion was switched to drug-containing solution. The holding potential was maintained at −80 mV. IQM-266 infusion was monitored with test pulses from −80 mV to +60 mV applied every 10 s until steady state was obtained (within 3 to 5 min) by using the clampex 11.1 utility of pClamp 11 software (Molecular Devices Co., San Jose, CA, USA). Other pulse protocols are described in the Results Section.

In order to calculate the block produced by IQM-266, the current was measured at the maximum peak and under the area of the current after applying different concentrations of the compound (0.001–500 μM). The percentage of block was calculated by:

% Block = 1 − (I_Drug_/I_Control_) × 100

From the fitting of these values to a Hill equation, concentration–effect curves were generated, obtaining the values of the IC_50_ and the Hill coefficient (n_H_).

Activation and inactivation were fitted to a monoexponential process with an equation of the form:

y = Ae^(−t/τ)^ + C

where τ represents the system time constant, A represents the amplitude of the exponential, and C is the baseline value. The voltage dependence of the activation and the inactivation curves were fitted to a Boltzmann equation:

y = 1/(1 + e^(−(−Vh)/s)^)

where s represents the slope factor, V represents the membrane potential, and V_h_ represents the voltage at which 50% of the channels are open or inactivated (activation or inactivation curves, respectively). In all cases, the control and the experimental condition was the same cell before and after being exposed to IQM-266 (see Results Section).

### 4.10. Statistics

Unless otherwise specified, data are expressed as the mean ± SEM. The number of biological replicates (n) is illustrated in the respective figure legends or graphs. The declared group size is the number of these biological replicates, and the statistical analysis was performed using these independent values. These independent cells were obtained from, at least, 5 different transfections. Direct comparisons between mean values in control conditions and in the presence of drug for a single variable were performed by paired Student’s *t*-test. Differences were considered significant if the *p* value was less than 0.05.

### 4.11. Materials

IQM-266 was dissolved in DMSO at a stock concentration of 5 mM. Further dilutions were performed in external solution that was constantly superfused at a rate of approximately 1 mL/min in the bath onto the cells at the desired concentration by gravity. Unless specified otherwise, drugs and general reagents were obtained from Sigma-Aldrich (Madrid, Spain).

## Data Availability

The data that support the findings of this study are available from the corresponding author upon reasonable request.

## Supplementary Materials

The following supporting information can be downloaded at: https://www.mdpi.com/article/10.3390/ijms23169170/s1.
