# Supplementary material for: Modulation of KV4.3-KChIP2 Channels by IQM-266: Role of DPP6 and KCNE2
Source: Int J Mol Sci. 2022 Aug 15;23(16):9170. doi: 10.3390/ijms23169170 (PMC9409462; doi:10.3390/ijms23169170)
Supplement: Supplementary file 1 [file ijms-23-09170-s001.zip › ijms-1859614-supplementary.pdf]

Modulation of KV4.3-KChIP2 Channels by IQM-266: Role of DPP6 and KCNE2

Angela de Benito-Bueno [1,†], Paula G. Socuellamos [1,†], Yaiza G. Merinero [1], Pilar Cercos [2], Carolina Izquierdo [2], Miguel Daniel-Mozo [3], Irene Marín-Olivero [4], Angel Perez-Lara [4,5], Juan A. Gonzalez-Vera [4], Angel Orte [4], Armando Albert [3], Mercedes Martin-Martinez [2], Marta Gutierrez-Rodriguez [2,*] and Carmen Valenzuela [1,6,*]

[1] Instituto de Investigaciones Biomédicas "Alberto Sols" (CSIC-UAM), 28029 Madrid, Spain

[2] Instituto de Química Médica (IQM-CSIC), 28029 Madrid, Spain

[3] Instituto de Química Física Rocasolano, Consejo Superior de Investigaciones Científicas (IQFR-CSIC),

28006 Madrid, Spain

[4] Nanoscopy-UGR Laboratory, Departamento de Fisicoquímica, Unidad de Excelencia de Química Aplicada a Biomedicina y Medioambiente, Facultad de Farmacia, Campus Cartuja, Universidad de Granada,

18071 Granada, Spain

[5] Department of Neurobiology, Max Planck Institute for Multidisciplinary Sciences,

37077 Göttingen, Germany

[6] Spanish Network for Biomedical Research in Cardiovascular Research (CIBERCV), Instituto de Salud Carlos III, 28029 Madrid, Spain

[*] Correspondence: mgutierrez@iqm.csic.es (M.G.-R.); cvalenzuela@iib.uam.es (C.V.);

Tel.: +34-91-258-7493 (M.G.-R.); +34-91-585-4493 (ext. 4499) (C.V.)

† These authors contributed equally to this work.

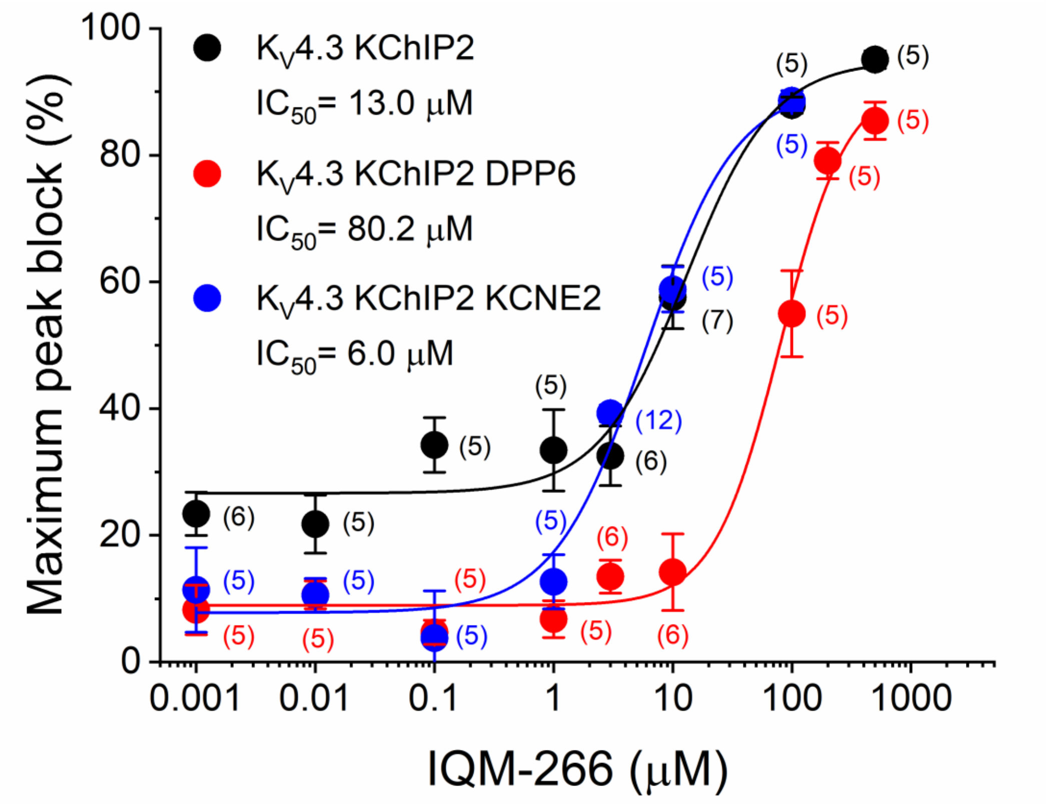

Supplementary Figure S1. Concentration-block of $K_V4.3$/KChIP2, $K_V4.3$/KChIP2/DPP6 and $K_V4.3$/KChIP2/KCNE2 channels induced by IQM-266. Each point represents the mean ± S.E.M. of, at least, 5 experiments.

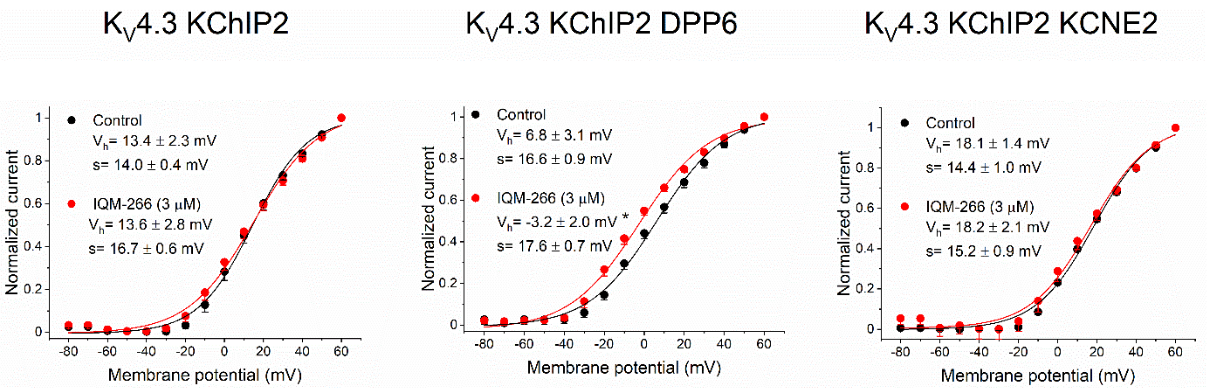

Supplementary Figure S2. Effects of IQM-266 (3 μM) on the activation curves obtained from the current-voltage relationships in the absence and in the presence of the compound (see Methods section) on K_V4.3/KChIP2 (n=9), K_V4.3/KChIP2/DPP6 (n=8) and K_V4.3/KChIP2/KCNE2 (n=7) channels in the absence and in the presence of IQM-266 (3 μM). *:P<0.05.

Funding: This publication is the results of the: Grants SAF2016-75021-R (to CV), RTI2018-097189-B-C22 (to MM) and BIO2017-89523-R (to AA) funded by MCIN/AEI/ 10.13039/501100011033 and by "ERDF A way of making Europe"; Grants PID2019-104366RB-C21 (to CV), PID2019-104366RB-C22 (to MGR), PID2020-114256RB-I00 (to AO and JAGV), PID2020-119805RB-I00 (to AA) funded by MCIN/AEI/ 10.13039/501100011033; Grant A-FQM-386-UGR20 funded by FEDER/Junta de Andalucía-Consejería de Transformación Económica, Industria, Conocimiento (to JAGV); Grant CB/11/00222 funded by Instituto de Salud Carlos III CIBERCV (to CV); Grants PIE202180E073 (to MM-M and MG-R), PIE201820E104 and 2019AEP148 (to CV) funded by Consejo Superior de Investigaciones Científicas. Grants BES-2017-080184 (to AB-B), BES-2010-036573 (to PC), PRE2018-083280 (to MD-M) and RYC2018-023837-I (to AP-L) funded by MCIN/AEI/ 10.13039/501100011033 and by "ESF Investing in your future"; Grant FPU17/02731 (to PGS) funded by Ministerio de Ciencia e Innovación.

Author Contributions: A.B.B., P.G.S. and Y.G.M. conducted the electrophysiological experiments in CHO cells and analyzed electrophysiological data supervised by C.V.; P.C. and C.I. synthetized IQM-266 supervised by M.G-R.; M.D-M. prepared KChIP2 protein supervised by A.A.; I.M-O. and A.P-L. performed tryptophan FRET experiments. A.P-L., J.A.G-V. and A.O. designed and supervised the binding experiments and analyzed data. M.M-M. conducted the computational studies. C.D. performed the electrophysiological experiments in mouse ventricular cardiomyocytes. A.B.B. and P.G.S. performed statistical analyses, generated the final figures and contributed to the manuscript writing. C.D., M.M-M., A.A., A.P-L., J.A.G-V. and A.O. contributed to the manuscript writing. M.G-R. and C.V. conceived the project, analyzed data, supervised the whole project and wrote the manuscript. All authors have read and agreed to the published version of the manuscript.

Conflict of Interest: The authors declare no conflicts of interest.

Data Availability Statement: The data supporting the findings of this study are available from the corresponding authors upon reasonable request. Some data may not be made available because of privacy or ethical restrictions.
